# Controversies concerning the diagnosis and treatment of bipolar disorder in children

**DOI:** 10.1186/1753-2000-4-9

**Published:** 2010-03-10

**Authors:** Erik Parens, Josephine Johnston

**Affiliations:** 1 The Hastings Center, 21 Malcolm Gordon Road, Garrison, NY, 10524, USA

## Abstract

This commentary grows out of an interdisciplinary workshop focused on controversies surrounding the diagnosis and treatment of bipolar disorder (BP) in children. Although debate about the occurrence and frequency of BP in children is more than 50 years old, it increased in the mid 1990s when researchers adapted the DSM account of bipolar symptoms to diagnose children. We offer a brief history of the debate from the mid 90s through the present, ending with current efforts to distinguish between a small number of children whose behaviors closely fit DSM criteria for BP, and a significantly larger number of children who have been receiving a BP diagnosis but whose behaviors do not closely fit those criteria. We agree with one emerging approach, which gives part or all of that larger number of children a new diagnosis called Severe Mood Dysregulation or Temper Dysregulation Disorder with Dysphoria.

Three major concerns arose about interpreting the DSM criteria more loosely in children than in adults. If clinicians offer a treatment for disorder A, but the patient has disorder B, treatment may be compromised. Because DSM's diagnostic labels are meant to facilitate research, when they are applied inconsistently, such research is compromised. And because BP has a strong genetic component, the label can distract attention from the family or social context.

Once a BP diagnosis is made, concerns remain regarding the primary, pharmacological mode of treatment: data supporting the efficacy of the often complex regimens are weak and side effects can be significant. However, more than is widely appreciated, data do support the efficacy of the psychosocial treatments that should accompany pharmacotherapy. Physicians, educators, and families should adopt a multimodal approach, which focuses as much on the child's context as on her body. If physicians are to fulfill their ethical obligation to facilitate truly informed consent, they must be forthcoming with families about the relevant uncertainties and complexities.

## Introduction

In September 2007, a group of researchers made headlines when they reported a forty-fold increase in the number of office visits in which children had a diagnosis of bipolar disorder (BP)[1]. The researchers estimated that whereas, in 1994-1995, in about 25 out of every 100,000 visits a child had a bipolar diagnosis, the number increased to 1,003 per 100,000 by 2002-2003. During the same ten-year period, office visits by adults with a BP diagnosis almost doubled from 905 to 1,679 per 100,000 annually, suggesting that BP diagnoses reported by community-based clinicians have increased across the age span. But the very low base rate of this diagnosis in youth coupled with a rapid rise signaled a major practice change.

Once thought rare in pre-adolescents, BP is now increasingly diagnosed in children, including preschoolers [2]. The drugs used to treat it include mood stabilizers and antipsychotics [3], which carry the risk of significant side effects. Perhaps even more than the diagnosis and treatment of Attention-Deficit/Hyperactivity Disorder (ADHD) and childhood depression before it, the ascension of the BP diagnosis in children and its treatment with medications whose risk/benefit profiles are inadequately established have generated debate in both lay and professional communities.

This commentary grows out of a workshop that explored the debates regarding the increase of BP diagnoses in children under 17. The workshop was the third of five in a series aimed at exploring the controversies concerning the diagnosis and treatment of mood and behavioral disturbances in children; the workshop was highly interdisciplinary, including child psychiatrists, psychologists, philosophers, sociologists, anthropologists, and others. Our first commentary, which grew out of our first workshop, explored the debates in general [4]. Our second commentary explained why informed people can disagree about ADHD diagnosis and treatment; we explored the "zone of ambiguity" between those children who clearly do--and those who clearly do not--have ADHD and the complexities of identifying and implementing effective treatment [5]. In this commentary, we focus on the intense and complex debate among child psychiatrists and psychologists about how best to conceptualize the serious emotional and behavioral disturbances exhibited by the group of children currently receiving a BP diagnosis.

This series of workshops was funded by a National Institute of Mental Health (NIMH) grant to The Hastings Center, which is an independent, nonprofit, nonpartisan, bioethics research institute. The authors of this commentary are scholars at The Hastings Center. One of us has a background in philosophical bioethics (EP) and the other a background in law and bioethics (JJ). Because neither of us has training in psychiatry, we relied on the generous advice of child psychiatrist Dr. Benedetto Vitiello at NIMH and former NIMH director Dr. Steven Hyman, who helped us identify a wide range of views within child psychiatry and psychology. We also relied on the generous advice of anthropologist Dr. Sarah Harkness to help identify individuals who study how social and cultural context can affect the interpretation of children's emotions and behaviors. Almost without exception, the experts we invited to the workshop accepted, even though we were able to offer only a very modest honorarium. We held the workshop, consisting of presentations and lengthy follow-up conversations, in New York City on April 24-25, 2008. (For more on our method, see [4]). *None of our advisors or workshop members, listed at the end of this commentary, bears any responsibility for its contents. This is not a consensus document*.

Our primary aim in this commentary is to fairly describe the debates that occurred at the workshop. As part of our effort to be as accurate as possible, we will sometimes include workshop participants' exact words. Based on our analysis of the presentations and discussion at the workshop, as well as our reading in the professional literature, we conclude that the proposed new diagnosis of SMD, on which the Diagnostic and Statistical Manual (DSM) V's proposed diagnosis of Temper Dysregulation Disorder with Dysphoria (TDD) is based, may help to clarify the debate about a troubled group of children who are currently receiving a BP diagnosis but do not neatly fit DSM IV criteria. We will suggest that, though in the US a BP diagnosis can get children the treatment, school accommodations, and insurance reimbursements they desperately need and deserve, if applied too widely it can do more harm than good.

### Disagreement about labels, but agreement that these children desperately need help

Some researchers, physicians, and parents argue that the sharp increase in rates of BP diagnosis simply reflects overdue recognition of this disorder in children. As workshop participant and patient advocate Susan Resko urged us to remember, a 40-fold increase sounds like a lot until one recalls the raw percentages: from the BP diagnosis being present in 0.025% of office visits in 1994 to it being present in approximately 1.0% of the visits in 2003. Moreover, these percentages refer to a clinic sample, not the community at large.

Those who are not alarmed by the increase suggest that in the past clinicians missed cases of BP because they did not understand that it can affect children and because BP symptoms can look different in children and adults. As child psychiatrist David Axelson asked, "if it is possible for children to suffer from anxiety, depression and other disorders experienced by adults, why not BP?" Moreover, they emphasize that, untreated, these children risk school failure, rejection by peers, physical injury, substance abuse, and even suicide--and their families can be torn apart.

Others at the workshop argued that BP in children is poorly defined, which can lead to misdiagnosis and inappropriate treatment. While children can have BP, they maintain that it is extremely rare and that when it is present the symptoms are very like those observed in adults. The recent increase in BP diagnoses in children is due to a redefinition of mania, key to BP diagnosis. Critics of this development hold that children are now receiving the BP diagnosis instead of one or more of the diagnoses they might have received in the past (e.g., ADHD, oppositional defiant disorder [ODD], and conduct disorder [CD], learning disorders, and pervasive developmental disorders [PDD])--or instead of some altogether new diagnosis (e.g., SMD). Some critics suggested that the BP diagnosis can be more palatable to some parents, teachers, and physicians than other better-fitting diagnoses, because the BP label attributes the child's problematic moods and behaviors to what is perceived to be a context-independent, genetic disorder. Such critics suggest that the publication in 2000 of *The Bipolar Child *by Dimitri and Janice Papolos [6] and a 2002 *Time *magazine cover story [7] precipitated a surge of parents asking physicians to give their troubled children the BP diagnosis.

Critics are also concerned about the medications used to treat BP. They observe that these drugs may not help the child, may cause harmful side effects, and increase the risk that other measures will be overlooked. Workshop participant and child psychiatrist Mary Burke speculated that, in the underprivileged community where she practices, one of the most effective ways to help children now receiving the BP diagnosis would be to promote attachment and reduce stress on families--stress that falls disproportionately on the poor [8,9]. Consistent with Burke's point about stress as a possible precipitant of the symptoms associated with BP and other diagnoses, is the research of workshop participant and child psychiatrist Boris Birmaher (and his colleagues), which suggests that low socio economic status is predictive of worse long-term BP outcomes [10].

While there was sometimes deep disagreement at the workshop about these points and others, there was universal agreement, including from those child psychiatrists who have been vocal critics of the way in which the BP diagnosis has been applied to children, that the children at issue desperately need help. As child psychiatrist Gabrielle Carlson said, psychiatrists agree that "there is a group of children with severe irritability or affective aggression or rages whose explosive behavior is significantly impairing, that we have been chasing with different diagnoses over the years, that populate child psychiatry clinics, and that we haven't had a great deal of success in treating." Regardless of which diagnostic label is ultimately applied, the children at issue experience extreme and often debilitating moods and exhibit deeply problematic behaviors, sometimes including suicidal and homicidal rages.

Clinical reports describe chronically impaired children who are highly irritable, angry, explosive and dysphoric, often as their baseline functioning [11-13]. They may also experience racing thoughts and periods of elation, grandiosity, hypersexuality, and suicidality [14]. Vivid portraits of the suffering of children diagnosed with BP (and of the suffering these children's symptoms can cause others) can also be found in some of the more detailed media reports, including the *Time *magazine cover story from 2002 [15] and a 2008 feature by journalist Jennifer Egan in the magazine section of the *New York Times *[16].

Psychiatric diagnoses are of course based on descriptions of clusters of behaviors that everyone in a population exhibits to some degree. Human beings, through our social institutions (in this case medicine), determine when clusters of these behaviors impair functioning enough to warrant a disease label. To emphasize the role of flesh-and-blood humans in reaching those determinations, workshop participant and anthropologist Emily Martin suggested that we might speak of "living under the description of" a given psychiatric diagnosis. Martin herself is diagnosed with BP and, because she fully acknowledges the very real impact her moods and behaviors can have on her ability to flourish, she seeks treatment for the disorder. Moreover, she is keenly aware that the cluster of behaviors that we call BP has been recognized for millennia and across cultures. But in saying that she "lives under the description of bipolar disorder," Martin emphasizes the respect in which these behaviors might have developed somewhat differently, been interpreted somewhat differently, and responded to somewhat differently in a different society or time [17]. Noticing the roles of interpretation and values in making psychiatric diagnoses should make us less surprised to see controversies about whether a given set of behaviors are bad enough to warrant a diagnosis and about what "the right" diagnosis is.

There is evidence that the pharmaceutical industry plays a distressingly large role in shaping those interpretations and values [18-20]. We note in particular the concern regarding financial conflict of interest recently raised about some BP researchers. While we share many of the concerns expressed in lay and academic publications about financial conflicts of interest and the role of the pharmaceutical industry in diagnosis development [21-25], exploration of these debates could not resolve the important questions regarding diagnosis and treatment of BP that were the focus of our workshop and are the focus of this commentary.

### The rate of BP diagnoses is rising faster in the US than elsewhere

Though debates about the diagnosis of BP in children can be traced back to the 1950s [26], and though Gabrielle Carlson published "Bipolar affective disorder in childhood and adolescence" in 1983 [27], the rapid increase in the number of diagnoses of BP did not begin in the US until the mid 1990s [1,28] (see Table 1). While some symptoms now associated with the BP label can be found in previous versions of the DSM in descriptions of disorders such as "unsocialized aggressive reaction of childhood," "adjustment reaction of childhood," and "schizophrenic reaction, childhood type," DSM's description of BP did not in the past and does not now explicitly address diagnosis of this disorder in childhood.

**Table 1:** Timeline: The Recent Debate about BP in Children

Early 1980s	Gabrielle Carlson et al. observe that bipolar symptomatology in preadolescent children can include severe irritability and emotional lability (as opposed to the classic symptoms that appear in adults and adolescents) [27].
1994	Geller et al. report in *Journal of American Academy of Adolescent and Child Psychiatry (JAACAP) *conversion to BP in 32% of a sample of children with major depression [31].

1995	Geller et al. in *Journal of Affective Disorders *suggested that children and adolescents with rapid-cycling mania characterized by elevated/expansive and/or grandiose mood have BP; the researchers did not use irritability to characterize BP because it also commonly appears in ADHD [29].

1995	Wozniak et al. [11] and Biederman et al. [30] in two *JAACAP *articles report that 16% of a clinical population of children met the criteria for mania primarily by exhibiting *chronically *irritable mood and describe their use of the Child Behavior Checklist to confirm their diagnoses of BP in these children.

1998	Klein et al., critique the move to consider chronic irritability a form of mania [47].

2000	Publication of *The Bipolar Child *by Demitri and Janice Papolos, a book aimed at parents, whose success coincides with an increase in visits to doctor's offices by parents regarding a bipolar diagnosis for their children [6].

2002	*Time *magazine runs a cover story on children with BP [15].

2003	Leibenluft et al. describe a new syndrome, Severe Mood Dysregulation, (SMD), which aims to bring some conceptual order to the increasingly heterogeneous class of children receiving a BP diagnosis [28,48,49,51].

2005	Article by Kowatch et al. describing treatment guidelines for children and adolescents with BP published in *JAACAP*. Accompanying commentary calls attention to lack of evidence that childhood diagnosis is contiguous with adult BP and critiques some symptoms as difficult to distinguish from developmentally normal childhood behavior [46].

2006	Brotman et al. use the label Severe Mood Dysregulation in the title of a scientific article, offering a new label for many children now receiving the BP diagnosis [49].

2007	*JAACAP *publishes practice parameter for diagnosis and treatment of BP in children and adolescents. Parameter follows Geller et al. diagnostic criteria more than those proposed by Biederman et al. but warns of difficulty differentiating symptoms from normal childhood behavior and urges periodic revisions of any diagnosis and treatment plan [57].

2007	*Archives of General Psychiatry *publishes study reporting a 40-fold increase between 1994 and 2003 in the number of office visits in which children (0-19) had a diagnosis of BP [1].

2009	Zito et al. report a 10-year trend for Medicaid-insured youth with clinician-reported pediatric bipolar disorder showing a proportional increase in minority youth with this diagnosis from 1997 to 2006 (23% increase in African-American and other minorities and corresponding drop in white youth) [85]. Crystal et al. report that poor children are four times more likely than wealthy children to receive atypical antipsychotics [70].

2010	Olfson et. al. report a doubling of the number of privately insured 2-5 year-old children with a psychiatric diagnosis who receive an antipsychotic, and lament the sparseness of non-pharmacological mental health resources [3]. Authors working on DSM V propose adding new diagnostic category, which is based on Severe Mood Dysregulation and may be called Temper Dysregulation Disorder with Dysphoria [54].

The increase in diagnoses seems to begin with germinal 1994 and 1995 articles by Barbara Geller et al., Janet Wozniak et al. and Joseph Biederman et al., which proposed that BP was more prevalent in children than previously thought [11,29-31]. When DSM-IV was published in 1994, it contained a new section, "Disorders Usually First Diagnosed in Infancy, Childhood, or Adolescence." While this section does not specifically mention BP, it does contain the observation that "many disorders included in other sections of the manual have an onset during childhood or adolescence [32]."

Since then, diagnoses of BP in children in the community have increased in the US [1,28]. Pre-school-age children are now among those receiving this diagnosis [2], which a short time ago was not thought to exist in early elementary school age youth (6-9 years) or even in early adolescence (10-14 years). In addition to calling attention to the increasing rate of diagnoses in children, critics have observed that the numbers are higher in the US than elsewhere and it seems that the US is the only country where BP is diagnosed in pre-school-age children. This situation suggested to workshop participant and child psychiatrist Jon McClellan (citing Soutullo et al. [33]) that something is askew in US diagnostic practices, and not, or not simply, in US genomes or environments.

While there are not good data comparing prevalence rates in the general population of children in different countries, the data comparing clinical populations (children brought to physicians' offices) reveal higher diagnostic rates in the US than many other nations. Psychiatrist and workshop member, Claudia Mehler-Wex, reported that, whereas 6% to 19% of children and adolescents in US clinical populations are diagnosed with BP [34-36], the diagnosis is virtually never made in England (1.7 cases/100,000/year)[37] nor Ireland (2.2 cases/100,000/year) [38]. Mehler-Wex reported that countries like Spain, India, Finland, Denmark, and Germany also fall well below the diagnostic rates of the US. (Brazil, where 7.2% of the clinical population is diagnosed with BP [39], is the only country with diagnostic rates close to the low end of the estimates for the US).

Mehler-Wex offered several reasons for higher rates of BP diagnoses in US children. First, DSM IV diagnostic criteria for BP cast a wider net and capture more affected individuals than do the International Statistical Classification of Diseases and Related Health Conditions 10^th ^Revision (ICD 10) criteria used in Europe. For example, where DSM IV requires only one episode of mania, or one episode of depression plus one episode of hypomania, to warrant a BP diagnosis, ICD 10 requires one episode of depression plus at least two episodes of mania [32]. Moreover, according to Mehler-Wex, practitioners in the US use lower thresholds for identifying symptoms than do their counterparts in Europe. Many of the children diagnosed with BP in the US would elsewhere be diagnosed with hyperkinetic disorder (roughly the ICD equivalent of ADHD), a different mood disorder, or a disruptive behavior disorder.

Another reason offered to explain higher diagnostic rates is that, as indicated by rates of stimulant and antidepressant use, US culture is in general more congenial than European cultures to psychiatric diagnoses and their pharmacological treatment in children. If, as psychiatrist Peter Kramer once suggested, the US was a country of "pharmacological Calvinists," it no longer seems to be [40].

On the other hand, workshop participant and child psychiatrist Joseph Biederman argued that nothing is "askew." His explanations for the difference in prevalence rates included: that Europeans are biased against recognizing psychiatric disorders in children; that Europeans and American reporting practices lead to differences in prevalence rates that are only apparent; and that US rates of diagnosis reflect a deeper understanding of the disorder among US psychiatrists.

### Diagnosing psychiatric disorders in children can be challenging

Before taking a closer look at the debates in the US, we should recall two reasons that it can be difficult to diagnose psychiatric disorders in children. Psychiatric disorders are predictable clusters of emotional, behavioral and sometimes somatic symptoms that cause impairment and emerge on a spectrum. Bright lines do not separate individuals whose emotions and behaviors are and are not disordered enough to receive a BP diagnosis. Second, because different diagnoses, some of which are themselves contested (e.g., CD, ODD, PDD, ADHD, and BP) can share some of the same symptoms, deciding which diagnostic label(s) to apply to a particular patient can be challenging.

Moreover, identifying symptoms and making a diagnosis can be harder in children than in adults. Younger persons can have difficulty noticing and describing symptoms and providing accurate accounts of time of onset and duration of symptoms (although children always have a secondary informant). Further, given how rapidly children's brains develop, even practitioners can and do disagree about whether a given behavior or mood is developmentally appropriate or a symptom of disorder. Is, for example, a 4-year-old's claim that she is superwoman a sign of imagination, self-confidence, or grandiosity? If a child accompanies her claim to be superwoman with a clear indication that she is about to jump from a hotel balcony, there is good reason to infer the presence of a symptom. Other times the answer will be less obvious.

In children and in adults, it can be tempting to conclude that a given medication's ability to reduce a particular symptom confirms a diagnosis, but it does not. Gabrielle Carlson offered the example of the atypical antipsychotic risperidone (Risperdal), which is effective at reducing aggression or rages in children diagnosed with BP--but is also effective in reducing aggression or rages in children with one or more disruptive behavior disorders (ADHD, ODD, and/or CD) and below average intelligence [41] as well as in children with autism [42]. Carlson suggested that atypical antipsychotics reduce rages the way aspirin reduces fever: "Regardless of whether the underlying cause is viral or bacterial, aspirin will reduce fever. But if the patient has a bacterial illness and the aspirin masks the symptoms temporarily, you'll think you've treated something you haven't. The patient won't get the antibiotic she needs."

### The DSM IV view of the BP spectrum

DSM-IV lists four BP subtypes: BP-I, BP-II, Cyclothymic Disorder, and BP-NOS [32]. In adults, the bar to getting the BP-I diagnosis is set fairly high and the classic symptoms of mania (even when mixed with depressive symptoms) are relatively easy for a well trained physician to identify. Much of the disagreement about diagnosing BP in children, however, revolves around determining just what mania looks like in children.

According to DSM IV, a full-blown Manic Episode entails "a *distinct *period of abnormally and persistently elevated, expansive, or irritable mood" lasting for at least 1 week. The central question in the pediatric debate is whether this episodicity criterion should be altered to include children who exhibit *chronic *irritability or cycle very rapidly between elevated mood and euthymia or depression. Under DSM, to meet the criteria for mania, when the patient's mood is *elevated or expansive *she must exhibit at least 3 of the following 7 symptoms: grandiosity, decreased need for sleep, pressure to keep talking, flight of ideas, distractibility, increased goal-directed activity, or excessive involvement in pleasurable activities that have a high potential for painful consequences. Alternatively, to meet the criteria for mania, when the patient presents with *irritability*, she must exhibit at least 4 of those 7 symptoms.

DSM sets the bar for BP-II lower in the sense that one does not need a full-blown Manic (or Mixed) Episode; having one or more episodes of Major Depression accompanied by at least one hypomanic episode suffices. (In hypomania the symptoms are the same as in mania, but their duration is shorter--4 days instead of 1 week--and they are less impairing.) The bar is lower still for Cyclothymic Disorder because one only needs numerous periods of hypomanic symptoms and periods of depressive symptoms that do not meet criteria for Major Depressive Disorder. The Cyclothymic Disorder label, however, is rarely applied to adults or children. Finally, to receive the BP-NOS diagnosis, one does not need to meet the criteria for any of the preceding 3 subtypes of BP. For example, one may have an abnormal mood, constituted by a rapid alteration between manic and depressive symptoms, but those symptoms do not meet the minimal duration criteria for a Manic Episode or Major Depressive Episode.

Workshop discussion and debate focused not on diagnosis of those rare children who exhibit discrete episodes of mania and meet full DSM criteria for BP-I, but on whether the majority of the children described by researchers like Geller et al. and Biederman et al. were best captured by the BP label or by some other diagnosis. Child psychiatrists' thinking about these difficult-to-diagnose children has evolved in, very roughly speaking, three stages since 1994.

### Stage 1: Expanding the definition of BP

Geller et al., Wozniak et al., and Biederman et al., were not the first to challenge the view that mania is rare in children, but their 1994 and 1995 papers have proved highly influential.

In 1994, Geller et al. reported that 32% of a sample of 79 children diagnosed with major depression had converted to BP-I or BP-II when followed over a 2-5 year period [31]. The following year, Geller et al. reported diagnosing 26 children aged 7-18 years with BP using a semi-structured diagnostic interview instrument [29]. They sought to define BP in a way that would allow them to cleanly distinguish it from ADHD: because one of the cardinal symptoms of mania--irritability--is also a symptom of ADHD, they would not give a BP diagnosis to children who exhibited only irritability. On their approach, for mania to be present (and for a BP diagnosis to be made), children had to exhibit elevated or expansive mood or be grandiose. Crucially, they also maintained that manic and hypomanic symptoms look different in children than in adults. Specifically, they modified DSM's criteria to allow a diagnosis of mania in children who *rapidly *cycled from mania or hypomania to euthymia or depression, including those who switched moods in the course of a day, and those whose symptoms did not have onset at the same time [29]. (Barbara Geller declined to participate in our workshop.)

At about the same time as Geller et al., were expanding or reinterpreting the DSM account of a manic episode characterized by *elevated *mood, Biederman et al. and Wozniak et al. were expanding or reinterpreting the DSM account of a manic episode characterized by *irritability*. In two 1995 papers, Wozniak et al. and Biederman et al. determined that 16% of their clinical population met the criteria for BP (they did *not *specify which BP subtype they observed)[11] primarily because of *chronic *irritability. They then argued that children who, based on a time-consuming structured interview, fully satisfied their understanding of DSM III criteria for mania, could also be identified with a relatively simple, cheap, easy-to-use symptom checklist, the Child Behavior Checklist (CBCL) [30]. While acknowledging the limitations of their study and the need for more research, Biederman et al. argued that, because "the clinical picture of pre-adolescent mania is very severe and impairing, there is a pressing need to *refine our methods *of diagnosing mania in such children (ital. added) [30]." (Critics of Geller's approach observe that 97.9% of the sample she reported in 1995 paper also exhibited irritable mood and that her later studies found rampant irritability in her sample [35,43], raising questions about whether Geller et al. and Biederman et al. are really observing different symptoms or are simply using different terms to arrive at the same diagnosis.)

To support his group's refined or expanded understanding of childhood mania, Biederman (citing Perlis et al. [44]) observed at our workshop that about 65% of BP adults report having BP symptoms as children or adolescents that were missed by their physicians. He therefore infers that BP symptoms can look different in children, and that clinicians can miss pediatric mania if they are looking for the classic adult presentation.

Many workshop members were not persuaded by either expanded conception of mania. Aside from concerns about reinterpreting the episodicity requirement, there were also concerns about whether the observed irritable or elevated moods were properly understood as symptoms of BP. Jon McClellan, for example, was critical of what was being counted as grandiosity, noting that "Normal children display numerous behaviors and beliefs that would be considered pathological by adult standards [45]." He also suggested that many of the children Biederman et al. diagnose with BP are just "moody kids with rage outbursts and aggression." Gabrielle Carlson observed that "euphoria is easy to find if you're hunting for it, and if you infer it from merely being silly"--as one could given some of the language in the 2005 treatment guidelines for BP in JAACAP [46]. She did, however, acknowledge that episodic euphoria--euphoria that represents a dramatic shift from that child's usual mood and that appears with other symptoms--may be easier to identify.

Biederman, however, argued that in its intensity, frequency, and association with "out-of-control aggressive behavior," the irritability associated with BP is "qualitatively distinct" from the irritability associated with other childhood disorders (such as ADHD or CD). He argued that, much like a neurologist can determine the difference between a seizure associated with petit mal and one associated with temporal lobe epilepsy, so can a properly trained child psychiatrist distinguish between the different types of irritability associated with different diagnoses.

Rachel Klein argued, as she had in *JAACAP *in 1998, that it is a mistake to interpret chronic irritability as mania [47]. For irritability or elevated mood to count as a symptom of BP, it must appear in distinct and sustained episodes--not as chronic or rapidly cycling. As Klein put it, episodicity is a *sine qua non *of BP.

### Stage 2. Tightening the definition of BP and beginning to define a new diagnosis

In the early 2000s, some researchers began to speak of a BP spectrum, stretching from Narrow Phenotype BP to Broad Phenotype BP. Narrow Phenotype children were largely synonymous with strictly defined BP-I patients. In 2003 Ellen Leibenluft et al., described the Broad Phenotype children:

Children exhibiting the broad phenotype may ultimately prove to be a heterogeneous group. Some may eventually meet the strict criteria for (hypo)mania; the course of others' illness may be consistent with dysthymia, major depressive disorder, or some form of disruptive behavior disorder; and still others may prove to have a syndrome that is not well captured by the current diagnostic system [48].

That is, while there was a small group of children who did warrant the Narrow Phenotype BP (or BP I) label, there was a larger group that would be better be captured under the rubric of Broad Phenotype BP. Some of the children in that latter, heterogeneous group might someday exhibit BP I, some might be conceptualized as exhibiting depression or a disruptive behavior disorder--and some might best be conceptualized as having a disorder that was not articulated in DSM IV. That is, Leibenluft et al. were suggesting that some children who had been receiving a BP diagnosis might be better served by a new diagnosis, perhaps called Severe Mood Dysregulation (or, as the DSM V editors are currently proposing, "Temper Dysregulation Disorder with Dysphoria") [39].

### Stage 3: Severe Mood Dysregulation category gains support

As others became familiar with the SMD label, and as the data showing differences between SMD and BD grew stronger, Leibenluft et al. largely dropped the Broad Phenotype BP label. In 2006, SMD appeared for the first time in the title of a scientific article [49], describing a group of children who *share *severe irritability and hyperarousal symptoms with BP I children, but who exhibit *chronic *irritability and do *not *share the hallmark elevated mood or grandiosity required by the DSM diagnosis of BP I. These children were said to exhibit developmentally inappropriate reactivity to negative emotional stimuli, such as "outbursts characterized by yelling and/or aggression [28]," which occur at least 3 times a week, and are impairing in at least 2 settings (home, school, peers). To receive the SMD syndrome label, children must experience symptoms for at least a year without more than 2 symptom-free months. Onset begins before age 12 (according to Leibenluft et al.'s data, average age of onset is 5.1 years) (personal communication). A prevalence study by Brotman et al. found that SMD is relatively common in children, with a prevalence rate of 3.3% [49].

Children with SMD also exhibit ADHD- and mania-like symptoms, including 3 of the following: insomnia, intrusiveness, pressured speech, flight of ideas/racing thoughts, distractibility, and psychomotor agitation [28]. But the severity of the irritability and the intensity of mood/anxiety symptoms are greater in youths with SMD than the average child receiving the ODD and ADHD diagnoses. Moreover, the ADHD plus ODD diagnosis fails to capture the mood and anxiety symptoms that characterize SMD youths.

At our workshop, Leibenluft began to sketch the argument that SMD meets the Robins and Guze criteria for a valid diagnosis [50]. SMD children are at significantly increased risk for developing depression--not BP--at age 18 [49]. These children are less likely to have parents with BP than are children with BP [51]. When playing frustrating games, the brains of children with SMD and BP respond differently (as measured by EEG) [52]. A new study by Brotman, Leibenluft, and others, using fMRI, finds that children who have received the BP, SMD, and ADHD diagnoses exhibit unique neural correlates in emotion processing of neutral faces [53].

Some challenges were put to Leibenluft at the workshop. Biederman argued that no new nosological entity is needed because the ADHD and ODD diagnoses together adequately capture the children Leibenluft et al. are classifying as SMD. Sociologist Ilina Singh asked if part of Leibenluft's argument was circular because, by invoking emerging neuroimaging data purporting to show that the brains of children with SMD and BP function differently [28], the assumption is made that we already know just what we are trying to figure out: what SMD is. Leibenluft responded pragmatically: "You have to start somewhere. Start with observation, test, now look at brains, refine categories. It's an iterative process"

Gabrielle Carlson, who has long treated and written about children with the constellation of symptoms at issue, believes that, in addition to recognizing the difference between SMD (or BP, for that matter) and ADHD, physicians also need to recognize the possibility that children who receive one of those diagnoses might actually have a learning disorder or a PDD spectrum disorder. Such children, Carlson argued, exhibit the sorts of aggressive rages that Leibenluft links with SMD (and that Biederman links with BP). While largely agreeing with Leibenluft et al., Carlson was stressing how easy it is to miss a learning disorder or a PDD spectrum disorder, among others. In a similar vein, Mary Burke voiced the concern that some children diagnosed with BP might more helpfully be diagnosed with PTSD or what she calls "parent-child relationship disturbance" (PCRD), which in her clinical experience are often overlooked.

We recognize the magnitude of the contributions made by Biederman et al. and Geller et al.: with their research they have brought much needed attention to a group of deeply troubled children. "Diagnostically homeless children," as Carlson calls them, can be very difficult to help and to get help for from educational, medical and other support systems. Because of the way mental health and special education services are currently funded in the US, an ill-fitting diagnosis can be more helpful than no diagnosis in securing services (such as hospitalization and prescription medications) as well as disability status and other support services. Researchers, too, usually need a DSM diagnosis if they hope to find funding for their research. (We note, though, that NIMH is keenly aware of, and attempting to help researchers deal with, the ramifications of this nosological debate.) Despite these pragmatic concerns, however, we were persuaded that departing from a narrow interpretation of the DSM criteria risks confusing the discussion about the nature of the mood and behavioral problems suffered by the children at issue. We believe that a new diagnosis, along the lines of SMD, could prove more helpful to children, families, physicians and researchers. Indeed, it was recently announced that either SMD or TDD is being considered for inclusion in DSM V [54].

### Why does the diagnostic label matter?

Overall treatment recommendations, monitoring, and prognosis can be different for a child diagnosed with BP and a child diagnosed with, say, ADHD, a learning disability, or PTSD. However, because the medications used to treat these different diagnoses can also be the same, one might ask: what difference does it make which diagnosis a child receives?

Gabrielle Carlson responded that even if many of the same medications are prescribed for BP and some of its diagnostic cousins, the overall treatment plans and prognoses for the children are different. For example, stimulants can trigger mania in people with BP [55], and there is evidence that antidepressants can also [56]. Conversely, children who actually have ADHD, depression, or anxiety and who are treated with the standard BP medications may experience the side effects of those medications and not improve. Moreover, because DSM's diagnostic labels are meant to facilitate research, applying them inconsistently can compromise it [57].

As Carlson also emphasized, focusing on BP can "blind clinicians to the fact that there are other things they might be focusing on." That is, because BP is associated with high heritability estimates and is treated primarily with medications, physicians may (erroneously) infer that psychosocial treatments will not be helpful, or may be less inclined to delve deeply into the quality of the child's home environment or family relations.

### Complexities surrounding pharmacological treatment

As indicated by the 2007 practice parameter that appeared in *JAACAP*, the first mode of treatment for children with strictly-defined mania is a combination of drugs, including traditional mood stabilizers such as lithium, anticonvulsant mood stabilizers such as divalproex (Depakote) and carbamazepine (Tegretol), and the newer, "atypical" antipsychotics such as olanzapine (Zyprexa), quetiapine (Seroquel), and risperidone (Risperdal). However, there are virtually no published research studies evaluating either the long-term (i.e., longer than 6 months) effectiveness or the safety of these pharmacological combinations. Support for their use is based on studies of individual medications or, in rare instances, on adjunctive treatment.

Moreover, according to some workshop participants, the efficacy of some individual medications used to treat children with BP is either unimpressive or not yet adequately established. Workshop participants Gabrielle Carlson and Julie Zito assessed the data on the efficacy of the mood stabilizers lithium, divalproex, and carbamazepine in treating children as "weak." That is, response rate (the ability to reduce symptoms of mania by 50%) in these medications did not beat placebo. Response rates for atypical antipsychotics show a better, 60-80% response with monotherapy for treatment of acute mania or mixed episodes compared to about a 25% response to placebo [58].

As the authors of the 2007 *JAACAP *practice parameter lament, due to limited studies in youth, "most of the treatment recommendations for early-onset BP are derived from the adult literature [59]." Of the 18 or so medications routinely prescribed for the treatment of BP, the FDA has approved only four for use in children, specifically: lithium for children over 12 years old, risperidone (Risperdal) [60] and aripiprazole (Abilify) for children over 10, and haloperidol (Haldol) for children over 3. (In addition, an advisory panel to the FDA recently recommended approval of quetiapine [Seroquel] and olanzapine [Zyprexa] for children over 13 years, although official FDA approval has not yet been made.) All other medications are used off label, including when approved medications are used to treat children younger than the specified age limits. While off-label use of medications is common in medicine, including in children, it is far from ideal. Experience with other medications has shown that because children are physiologically different from adults, medications that are generally safe and effective in adults are sometimes unsafe or ineffective in children [61-63].

Because many children with BP can also have another diagnosis (or diagnoses) such as ADHD, depression, anxiety, ODD, or OCD, additional medications may be added to the drug regimen, including antidepressants, stimulants, and first-generation antipsychotics such as haloperidol (Haldol) and chlorpromazine (Thorazine). And because every medication can have side effects, still more medications can be added to the regimen to treat the side effects.

The side-effects problem is as worrisome as it is familiar. Atypical antipsychotics are associated with extreme restlessness, uncontrollable speech and involuntary movements, and to a lesser degree tardive dyskinesia as well as drowsiness, increased metabolic problems, and significant weight gain [64-66]. The latter side effect creates additional risks, including juvenile diabetes and high cholesterol [57]. Mood stabilizers such as lithium and anticonvulsants also carry the risk of significant weight gain, drowsiness, and decreased cognition, as well as the development of tremors. Many of these medications carry risks to fetuses, leading the *JAACAP *practice parameter to recommend that clinicians perform adjunctive pregnancy tests and specifically warn female patients and their families about concerns regarding the anticonvulsant valproate and polycystic ovary disease [57,67,68].

Pharmacoepidemiologist Julie Zito pointed out that, while the complicated and difficult symptoms associated with BP call for complex treatment regimens, we know that, as a general rule, "the larger the number of medications used to treat a condition, the greater the risk of adverse events [69]." Yet, as Mary Burke suggested, "treatment guidelines support polypharmacy," as do the realities of clinical practice. "If you only have 20 minutes a month [with a child]" she argued, "it is easy to add a second antipsychotic." Joseph Biederman, however, offered reasons to explain and defend polypharmacy, including: children with BP often also have other disorders and therefore require more than one medication; one drug alone may not be as effective as that drug in combination with an additional drug(s); and additional drugs are sometimes needed to treat side effects of another effective but poorly tolerated medication.

As the number of BP diagnoses in children has increased, so has the number of prescriptions in at least two of the drug classes listed above: anticonvulsants and atypical antipsychotics. In a 2009 article in *Health Affairs*, Stephen Crystal et al. describe not only sparse data regarding the efficacy of the newer or "atypical" antipsychotics and plentiful data regarding their metabolic risks, but they also describe a trend whereby low-income American children are as much as four times more likely than higher-income children to receive atypical antipsychotics (for BP and other psychiatric diagnoses) [70]. As Crystal et al. also say, however, no one knows at this point why poor children receive treatment with antipsychotics at such a high rate. One possible explanation has to do with social control, or an unwillingness to invest in costly non-pharmacological approaches for poor children. Another explanation would observe that because poorer children are subjected to greater stress than wealthier children, their symptomatology may be worse [71], which may make more intensive pharmacotherapy appropriate.

Julie Zito presented community-based population data at the workshop showing between 2- and nearly 6-fold increases in the use of anticonvulsants by children aged 0-19 years across a 10-year period [72]. Anticonvulsants of course also have non-psychiatric uses, but in one study Zito and colleagues reported that 81% of youth who received an anticonvulsant-mood stabilizer were prescribed it for a psychiatric diagnosis and only 19% had a seizure-related diagnosis [73].

Zito also presented demographic data showing that children taking anticonvulsants are increasingly under 13 years old, male, and African American (14% in 2004-2005 compared with 6% in 1996-1997) [74]. The same data show that 50% of the children taking anticonvulsants have a BP diagnosis, and that 38% received a stimulant, 40% an antipsychotic, 52% an antidepressant, and 12% another psychotropic medication (including lithium) in addition to the anticonvulsant. Zito et al. found that in 2004/2005 pediatricians and other non-psychiatry specialists wrote a larger proportion of anticonvulsant-mood stabilizer prescriptions for youth with psychiatric diagnosis than in the previous decade [75].

When Gabrielle Carlson asked the deceptively simply question, "Do we know if, after taking these drugs, these kids are better off?" Julie Zito answered, "We have little data on the effectiveness of treatment in community populations. I can tell you about risks from some post-marketing systems, e.g. the FDA Adverse Event Reporting System, but there is insufficient evidence of benefits in community-treated populations." Zito herself then asked, "When will we get serious about outcomes by developing the infrastructure, design and measurement protocols to provide the benefit and risk information we need to assess medication outcome in community populations [76]?"

Of course, against the potential side effects and the less-than-ideal efficacy of these agents must be weighed the risks of not treating children with prescription medications. When a child's moods and behaviors are causing her significant distress and are impairing her ability to learn, develop friendships, and participate in family life, physicians and parents may decide that medication treatment is the only, or is an important, way to stabilize the child so that her well-being can be preserved or addressed through psychosocial or educational interventions. Many workshop participants, including those critical of current prescribing practices for BP, agreed that there are times when not medicating a child carries serious risks.

Our goal here is not to assess the validity of the evidence for any drug or treatment approach. Instead we seek simply to emphasize that the facts are not as complete as families and physicians would wish them to be. Prescribing medication is always a balancing act, with physicians and parents weighing what is known about the drug's effectiveness and side effects against the severity of the symptoms the medication will target. In the case of the drugs used to treat BP (and related disorders) the balancing act can be difficult due to a lack of agreement about the diagnosis and a lack of information about the safety and efficacy of the medications. Physicians and other mental health care professionals have an ethical obligation to be honest with parents about these complexities. Policy makers, funders, and researchers have an ethical obligation to ensure that research is funded and conducted to fill these knowledge gaps.

### Psychosocial treatment

There was agreement at our workshop, as there is in much of the literature, that medications will often be the first-line treatment for children diagnosed with BP or presenting the specific symptoms discussed here. However, many workshop participants also stressed that psychosocial treatments can complement pharmacotherapy, and they lamented a lack of attention to these treatments. Child psychologist David Miklowitz quipped, "We're getting to the point where psychosocial treatment is being called non-pharmacological treatment."

The workshop members who spoke about "non-pharmacological" treatments spent little time trying to distinguish among closely related diagnoses. Instead, they described different psychosocial interventions, summarized what is known about the effectiveness of these interventions at changing behavior and assisting children and families to cope with difficult moods and behaviors, and described the barriers to greater availability of these treatments.

David Miklowitz, focused on four psychosocial treatments: family focused treatment (FFT), interpersonal and social rhythm therapy (IPSRT), Cognitive Behavioral Therapy (CBT), and psychoeducation. These therapies were selected in part because they have shown efficacy in adults with BP. He explained that two or more treatments are often best used in combination because those that focus on early recognition of prodromal signs and medication adherence affect mania more than depression and those that focus on interpersonal coping strategies affect depression more than mania. Miklowitz also stressed that treatments of three or fewer sessions do not work as well as treatments of twelve or more sessions.

One target of psychosocial therapies is stress management, because stress and trauma are both contributing causes to and results of manic episodes. Psychologist Mary Fristad emphasized environmental precipitants of mania, stressing what she labeled bi-directional causation. "If you have mania" she explained, "then you create a lot of trauma in your life and trauma precipitates [mania]." It is clear that early life stress can have a lifelong impact on neurochemistry, endocrine responsivity and behavior, and adult studies have shown that early manic episodes are more likely to be triggered by stressful life events than later manic episodes [77], all of which indicates the potential value of working with children and adolescents to manage stress and trauma. Stress can include anything from the expressed emotion of family members, peers, and teachers, to hypercriticality, to sexual abuse. Unfortunately, Fristad explained, particularly given the apparently strong genetic component of BP, parents who themselves have the disorder are at increased risk for being less attentive, less active, more over-protective, and more tense, which can create stress and trauma for their children.

Miklowitz presented data from several adult studies [78], including one that compared the effectiveness of each of FFT, IRST, CBT and psychoeducation in nearly 300 adults with BP [79]. He also discussed four studies in adolescents [8,80-82], one study of children and adolescents [83], and two studies in children [84]. In each, the study population was assigned to either psychosocial treatment(s) or a form of community or collaborative care. In all studies, the group of patients receiving one or more of the psychosocial treatment(s) was on average more likely to have (depending on the particular study's design) recovered from an acute episode of BP, experienced improvement in their levels of depression or mania, received a reduced score on a psychiatric rating scale, or improved on symptom measures. This data led Miklowitz to state that, as a rule of thumb, one or more psychosocial treatments "should accompany pharmacotherapy for early onset BP." (We are not aware of any studies that have attempted to discern whether some psychosocial interventions are more effective in children who receive the BP label versus those who receive the SMD label.)

Indeed, Miklowitz stressed that the studies cited above are just a beginning, and that more research is needed to better understand how, why, and when various therapies work. In particular, he argued that the goals of each treatment are not always clear. For example, which treatments are trying to modify treatment adherence, which family and peer relationships, and which the ability to recognize and act on prodromal symptoms? One barrier to such research is, according to Julie Zito, NIMH's failure to prioritize effectiveness research.

Cost is not only an issue in the context of research. Stephen Crystal et al. have observed that "nonpharmacological alternatives, which may involve teaching children problem-solving skills and teaching their parents to reward positive child behavior, are costly and difficult to disseminate [70]." Many workshop participants emphasized that this cost can effectively reduce the availability of psychosocial treatments and lead physicians and families to focus primarily or solely on medication. Psychosocial treatments need to be delivered by trained professionals, over a period of weeks or months, and are not always fully covered by medical insurance. Unlike appointments focused on medication management, psychosocial treatment appointments can last 30-50 minutes and may require active participation of multiple family members. While such treatments may significantly improve a child's symptoms and functioning, and while in the long term they may provide patients and their families with tools and strategies to manage and control symptoms without close supervision, in the short-to-medium term they are expensive and time consuming, requiring energy and commitment from all involved. NAMI representative Darcy Gruttadaro noted that " [while] families want more than medication, financial incentives and our stressed health care system favor writing scripts." To improve the accessibility of these therapies, health care reform must improve continuity of care and assure parity between mental health and medical services. These improvements are especially important for this difficult-to-treat population, regardless of the diagnostic labels we conclude are most helpful.

### Concluding observations

Children and families can suffer terribly as a result of the serious disturbances in children's moods and behaviors described here. Because moods and behaviors are distributed continuously in a population (without clean breaks between normal enough to leave alone and atypical enough to warrant intervention), both over- and under-diagnosis are likely problems, although we did not pursue these problems at this workshop. Instead, we focused on the controversies surrounding what "actual" BP looks like in children.

In the mid-1990s, researchers led by Joseph Biederman and Barbara Geller re-described the syndrome of mania, key to any BP diagnosis. As a result, children who exhibit, in the case of Geller et al., primarily rapidly cycling elevated/expansive and/or grandiose mood, or in the case of Beiderman et al., primarily chronic irritable mood, have received a diagnosis of BP. These conceptualizations of the disorder, in combination with other social and clinical factors, have fueled a significant increase in the number of children diagnosed with and treated for BP. Insofar as everyone agrees that some fraction of these children warrant the BP diagnosis, an increase in diagnostic rates is a good thing. The debate is about how large that fraction is.

Recently, Leibenluft and colleagues have proposed that many children currently diagnosed with BP may be better thought of as exhibiting a syndrome they call SMD. In addition, other researchers and clinicians have argued that a BP diagnosis may blind physicians to or mask the presence of disorders such as severe ADHD, CD, ODD, PTSD, PDD or some autism spectrum disorders. This year, the committee responsible for writing the next iteration of the DSM proposed the addition of a new childhood disorder to be called Temper Dysregulation Disorder with Dysphoria, which is based on Leibenluft et al.'s description of the SMD syndrome.

Based on our reading in the literature and discussion at our 2-day workshop, we (the non-psychiatrist authors) were persuaded that the BP label may fit poorly many (quantification is difficult) of the children who have received it over the last decade. We were also persuaded that, when DSM IV's criteria for BP are strictly applied to children, and psychiatrists revisit the diagnosis and treatment plan periodically, the results can be potentially life-saving and may reduce years of suffering for the child and family. Clearly, more research is needed to improve our understanding of the best way to conceptualize the relationships among the different clusters of symptoms that over the last 15 years increasingly have been captured with the label BP. DSM V appears to be addressing this challenge.

We understand that greater nosological clarity may be difficult to achieve, and that an ill-fitting diagnosis can sometimes be more helpful to children, families, and researchers than no diagnosis at all. It is a deeply regrettable feature of our current mental health and educational systems that some DSM diagnoses are better than others at getting children and families access to the care and services they so desperately need.

Additional clinician training may also be needed. Symptom checklists cannot substitute for thorough evaluations. Current training practices, as well as reimbursement policies, may leave some child and adolescent psychiatrists unable to deliver the "biopsychosocial" care that so many agree is the gold standard. In addition to funds for research and training, clinical and educational resources must be available to these children and their families to relieve immediate stress and to work towards long-term recovery, symptom remission, and reduced impairment. These resources represent a significant financial burden that cannot be borne by families alone.

Given the evolving state of the research, physicians making a BP diagnosis or treating children with a BP diagnosis must remain apprised of the debates and follow the AACAP practice parameter's recommendation to revisit the diagnosis and treatment plan at regular intervals. Though we appreciate the concern regarding "truth dumping" (where a physician shares an overwhelming number of partial facts with a patient), that concern should not prevent physicians from being honest with families. Although it may initially be distressing for patients and their families to hear that a diagnosis is not universally accepted and that treatment responses are debated, providing a false sense of certainty undermines the respect for persons necessary in the physician-patient relationship. Moreover, it may cause confusion and disillusionment in the future, if the diagnosis is revised or if treatment recommendations are altered.

If physicians are to fulfill their ethical obligation to facilitate truly informed consent, they must be forthcoming with families about the relevant uncertainties and complexities. This may not be easy, but it is essential. No one should be surprised that in such a massively complex arena of inquiry, there is uncertainty and disagreement even among the most well intentioned and well informed professionals.

## Conflicts of interests

The authors have no conflicts to claim. The workshop was funded by grant U13 MH78722 of the National Institute of Mental Health to the Hastings Center (Principal Investigator: Erik Parens, Ph.D.)

## Authors' contributions

Both authors contributed equally to this article.
